# INNOVATION DESIGN: MEDICAL DEVICE TESTING BEFORE DEPLOYMENT FOR RESEARCH STUDIES

**DOI:** 10.1093/geroni/igae098.2460

**Published:** 2024-12-31

**Authors:** Alyce Ashcraft, Donna Owen, Jessica Brashear

**Affiliations:** Texas Tech University Health Sciences Center, Lubbock, Texas, United States; Texas Tech University Health Sciences Center, Lubbock, Texas, United States; Texas Tech University Health Sciences Center, Lubbock, Texas, United States

## Abstract

Smart technology for point of care (POC) testing, allows the creation of new devices from a design innovation mindset. Refinement in POC testing for infection is profoundly needed because of continuing dissatisfaction with antibiotic stewardship. Determining optimal control conditions to detect when a device is working as expected is a first step.

**Aim:**

To describe the process of validating a device for POC infection assessment. A collaborative team involving Nursing, Clinical Laboratory Sciences, and Electrical Engineering drove device testing following a standard protocol validation for a POC instrument using American Society for Clinical Laboratory Science (ASCLS) guidelines. The protocol included comparison of two presumably identical devices relying on lasers for determination of specimen end points.

**Results:**

Differences between the device operation manual and expected performance indicators provided direction for engaging with the device design team. Functionality of devices was determined by noting operational differences. Validation failed for both devices. Multiple experiments were performed with controlled variables; one device provided expected results on 33% of runs, while the other only had expected results on 16% of its runs. A critical laser error was found in the instrument with the 16% success rate; mechanical issues with the other device were noted and have yet to be fully specified.

**Discussion:**

We engaged in collaborative ZOOM discussions, problem visualization screen shots, and photos of device elements. We learned more about the internal working of the device and proposed changes to the device to facilitate navigation around problems and begin the work of device redesign.

